# Supplementary material for: Context-dependent change in the fitness effect of (in)organic phosphate antiporter glpT during Salmonella Typhimurium infection
Source: Nat Commun. 2025 Feb 24;16:1912. doi: 10.1038/s41467-025-56851-5 (PMC11850910; doi:10.1038/s41467-025-56851-5)
Supplement: Supplementary file 1 — Supplementary Information [file 41467_2025_56851_MOESM1_ESM.pdf]

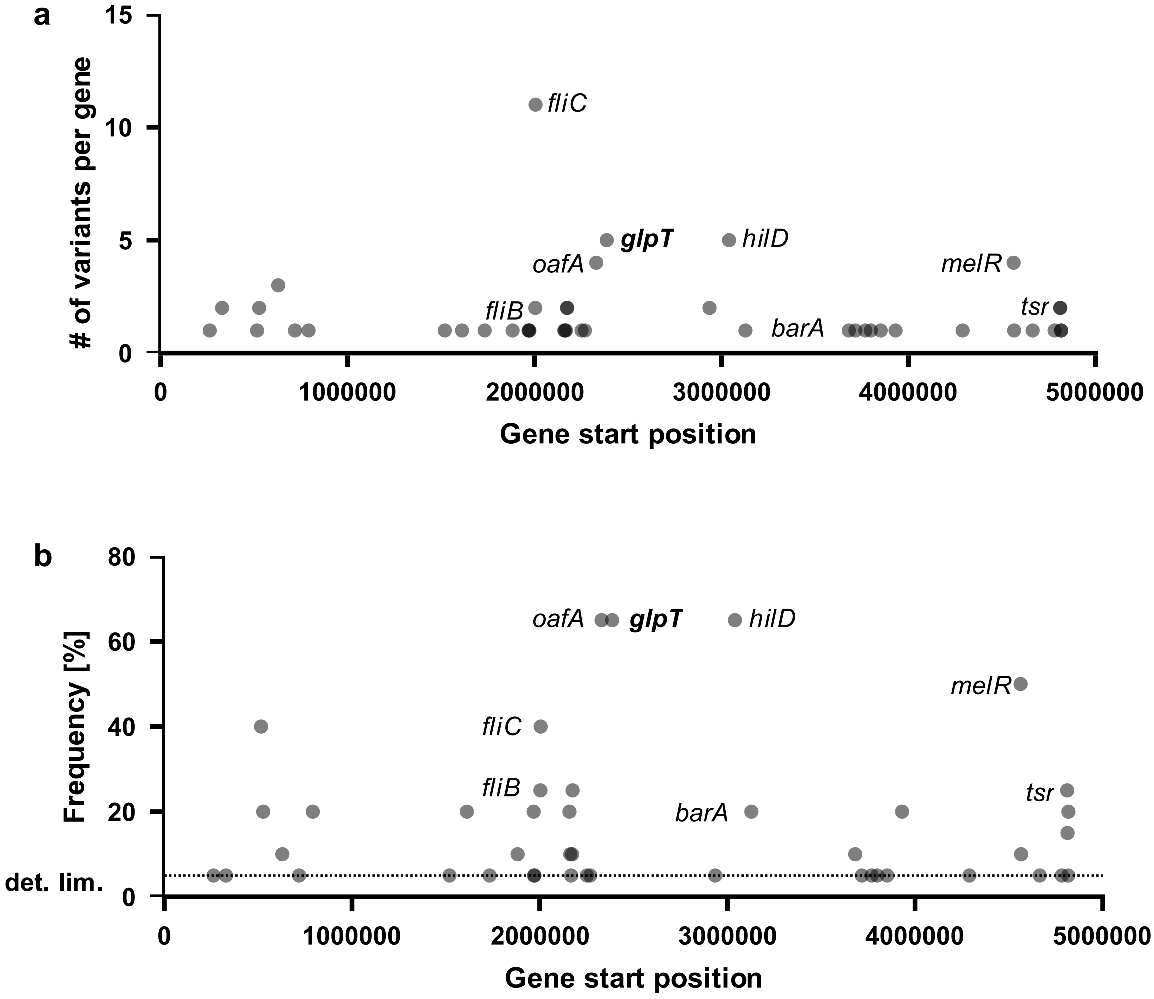

c	*Shigella*	*Escherichia*	*Enterobacter*	*Klebsiella*	*Cronobacter*	*Citrobacter*	*Salmonella*	n
Shigella	0.99							28
Escherichia	0.97	0.95						112
Enterobacter	0.93	0.90	0.96					83
Klebsiella	0.81	0.79	0.81	0.75				51
Cronobacter	0.92	0.90	0.93	0.80	0.98			12
Citrobacter	0.85	0.83	0.83	0.71	0.81	0.77		51
Salmonella	0.91	0.89	0.87	0.75	0.86	0.81	0.87	331

Figure S1 Conservation of GlpT in Enterobacteriaceae

a The number of distinct variants per gene for all genes that harbored mutations after 157 – 160 days of infection in Gül et al., 2023. The plot shows the gene start position in the genome and indicates that *glpT* is not part of a genomic region with an unusually high mutation frequency. Selected genes that have previously been identified to be subject to positive selection for their loss of function are indicated: *fliC* and *fliB*, required for motility (Cherry, 2020; Cummings et al., 2006; Parys et al., 2021); *oafA*, which encodes the O5-antigen (Hauser et al., 2011; Slauch et al., 1996); *hilD* (Cherry, 2020; Diard et al., 2013; Gül et al., 2023); *melR*, a positive regulator of the melibiose operon (Cherry, 2020); *barA*, which was recently shown to mutate in clinical *S. enterica* isolates (Grote et al., 2024); and *tsr* (Cherry, 2020; Gül et al., 2024).

b The frequency of isolated clones that harbored mutations in individual genes after 157 – 160 days of infection in Gül et al., 2023, and their gene start position in the genome. The *oafA* and *glpT* open-reading frames are 60 kb apart.

c The within- and between-group Sequence Identity matrix for Uniprot GlpT sequences of representative genera of the Enterobacteriaceae family shows that GlpT is highly conserved within Enterobacteriaceae. The last column in the graph displays the number of sequences that fulfilled the quality cutoff.

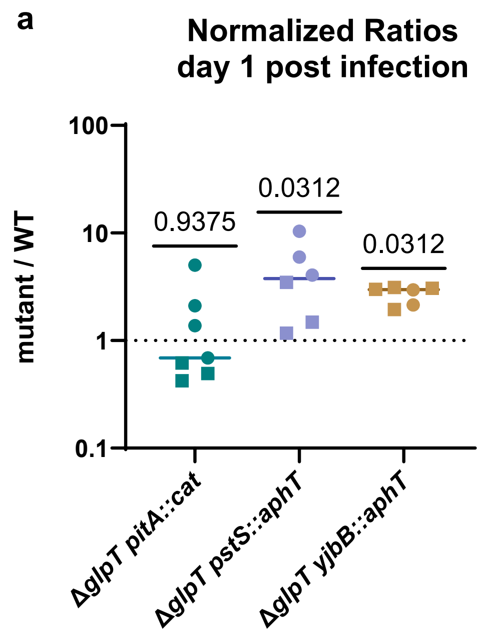

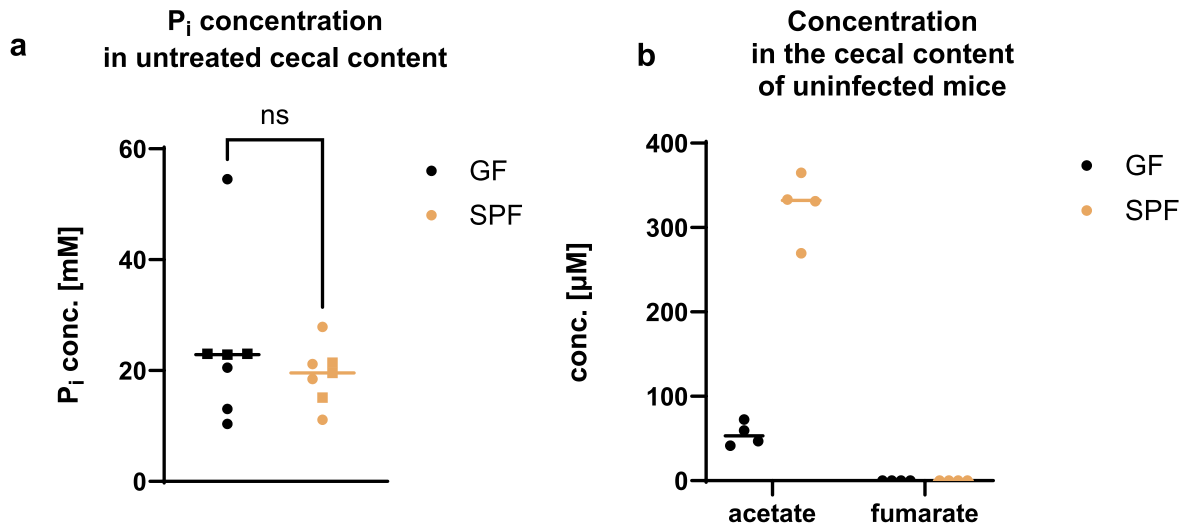

Figure S2 *glpT* competitive infections with strains deficient for a single P_i-transporter and validation of the P_i quantification protocol

a The concentration of inorganic phosphate in the cecal content of untreated germfree (GF) BL57/6J mice is comparable to the concentration in untreated specific-pathogen-free (SPF) mice. The line represents the median. The samples displayed as circles were additionally analyzed by liquid chromatography and mass spectrometry (shown in panel b). n = 7 mice.

b Acetate and fumarate concentrations measured in the cecal content of germ-free and specific pathogen-free (SPF) C57BL/6 mice indicate that our protocol, which was designed to avoid substantial lysis of microbiota cells, did not lead to such lysis. Lines represent the median. The concentration was quantified by targeted liquid chromatography and mass spectrometry on the samples displayed in panel a. The detection limits are 20 – 1250 µM for acetate and 12 – 750 µM for fumarate. n = 4 mice.

c Normalized ratios for the *glpT* mutant in the background of strains deficient for *pitA*, *pstS*, and *yjbB* against *S*. Tm WT, respectively. The statistical significance was tested using a one-sample Wilcoxon signed rank test against a hypothetical mean of 1 for a neutral competition. Lines represent the median. n = 6 for groups 1 and 2 and n = 7 for group 3 (*yjbB*).

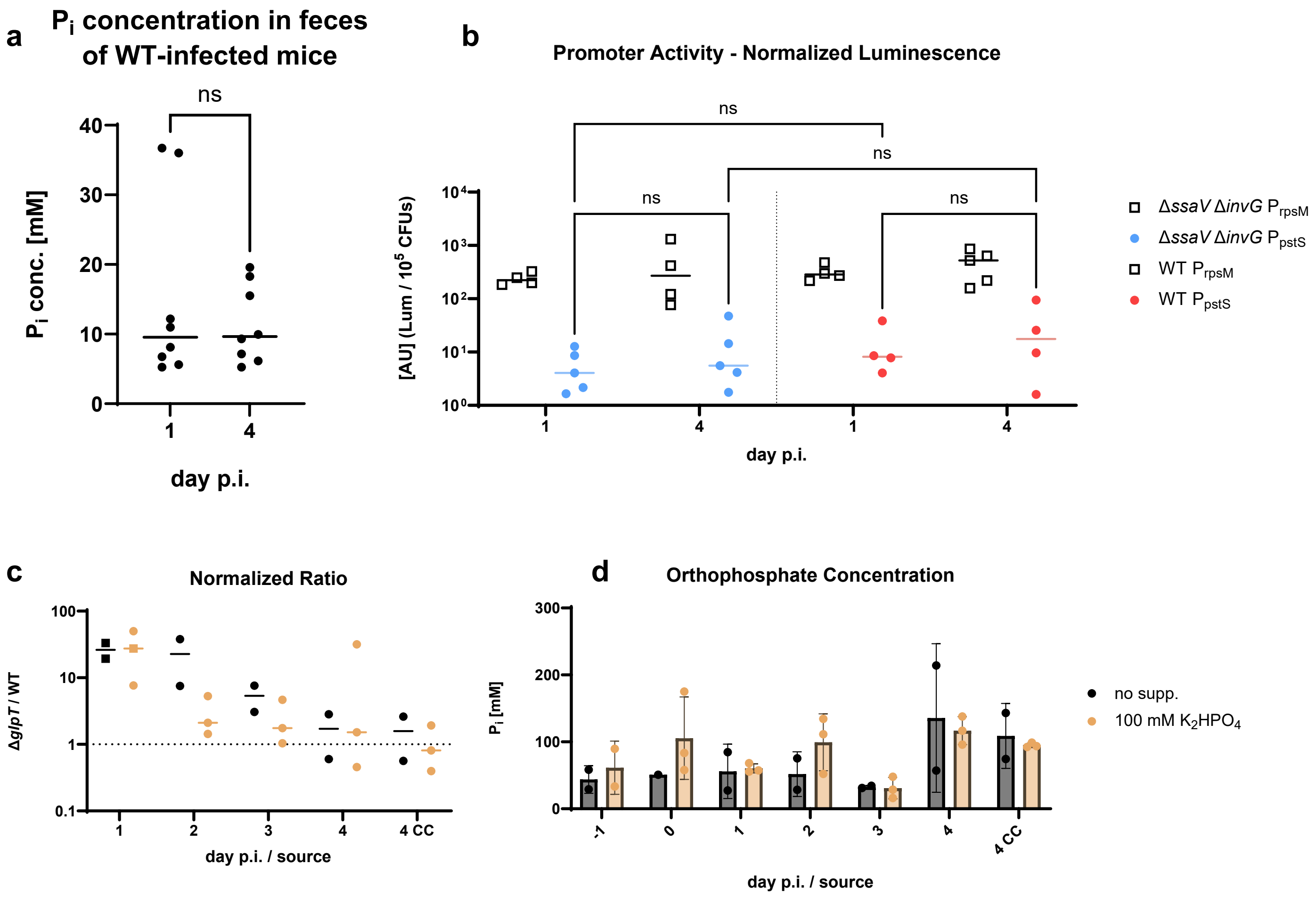

Figure S3 Orthophosphate and magnesium concentrations do not change over time or when they are supplemented in drinking water

a The phosphate concentration in feces does not decrease over time. Mice were strep-pretreated and infected with wild-type SL1344. The concentration was measured in feces, and statistical significance was assessed using a paired t-test. Lines represent the median. n = 8 mice.

b The promoter activity of the P_{pstS} reporter does not change over time or in the presence of inflammation. The constitutive promoter P_{rpsM} was used as a positive control. n = 4 mice for the P_{rpsM} reporter and the WT P_{pstS} strain and n = 5 for the Δ*ssaV* Δ *invG* strain carrying the P_{pstS} reporter.

c The orthophosphate concentration in feces is not influenced by supplementation of 100 mM K_2HPO4 in the mice's drinking water. The mice were pre-treated with streptomycin and infected with wild-type *S*. Tm. n = 2 mice without supplementation and n = 3 mice with supplementation in the drinking water.

d The ratio between the *glpT* mutant and the wild-type is not influenced by the addition of 100 mM K_2HPO_4 to the mice's drinking water. n = 2 mice without supplementation and n = 3 mice with supplementation in the drinking water.

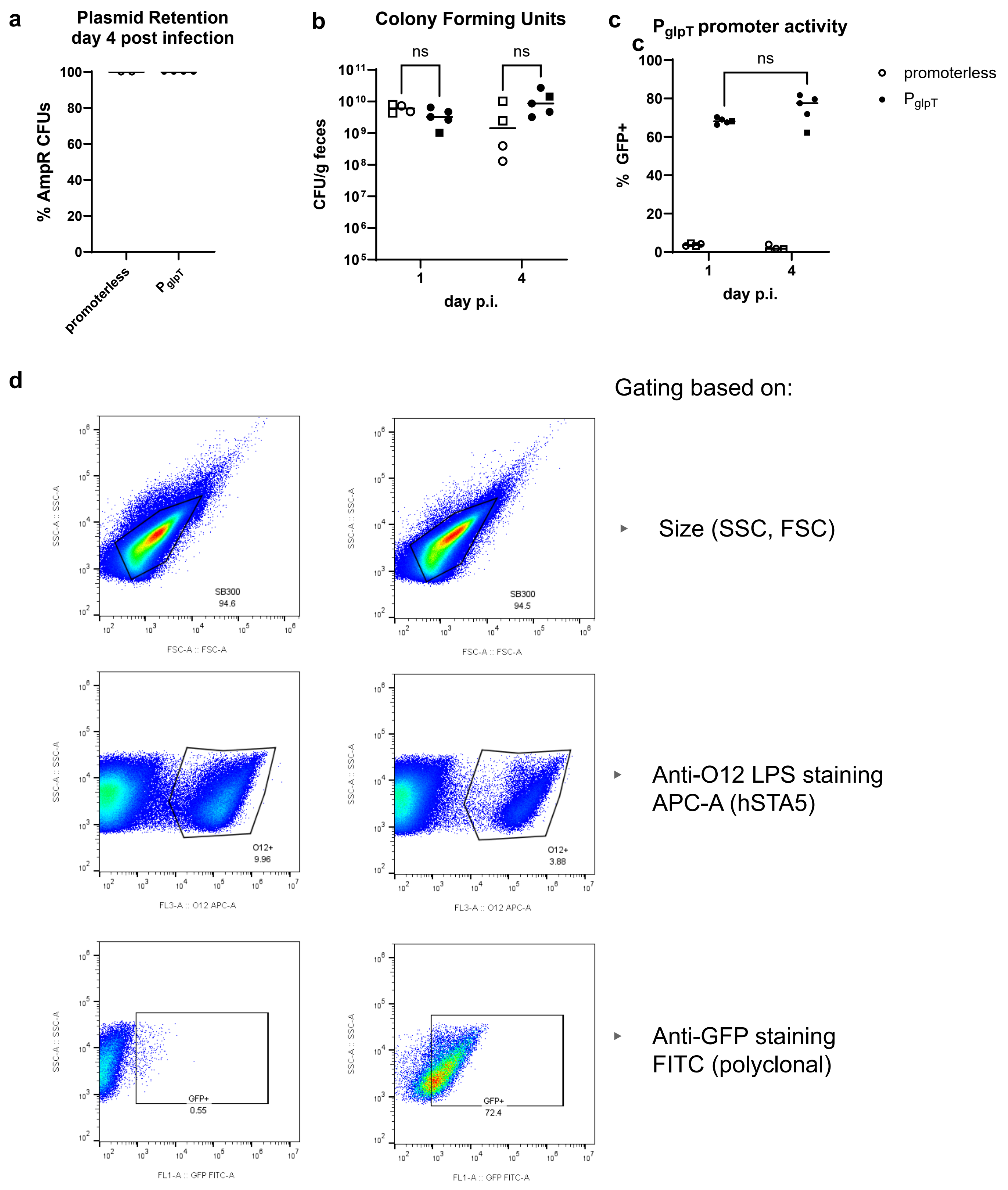

Figure S4 The fraction of *glpT*-expressing cells does not significantly change over time

a Plasmid retention was quantified for day 4 post-infection by replica plating from MacConkey Agar containing Streptomycin onto MacConkey Agar with Ampicillin and then Streptomycin. The fraction of cells resistant to Ampicillin and Streptomycin is shown for the samples displayed as circles in panels b and c. The lines indicate the median. n = 2 mice for the promoterless control and n = 4 mice for the P_{glpT} reporter.

b The colony forming units indicate that carrying the P_{glpT}-gfp reporter plasmid does not yield a significant fitness cost. The circles and squares represent the origin of the data points from two independent experiments. The horizontal lines represent the median. The statistical significance was tested with a two-way ANOVA (comparing strains and time points). n = 4 mice for the promoterless control and n = 5 mice for the P_{glpT} reporter.

c The P_{glpT} promoter activity does not change over time, measured as the gfp+ fraction of anti-O5 LPS-stained cells. The different shapes (circles and squares) indicate their origin from two independent experiments. The statistical significance was tested using a two-way ANOVA. Lines represent the median. n = 4 mice for the promoterless control and n = 5 mice for the P_{glpT} reporter.

d Exemplary images of the gating strategy used for the based on the cell size and shape, presence of lipopolysaccharide (to distinguish *S*. Tm cells from debris), and GFP fluorescence. Left panels were acquired for sample cells carrying a promoterless gfp construct, whereas the right panels were acquired for sample cells with the P_{glpT}-gfp reporter.

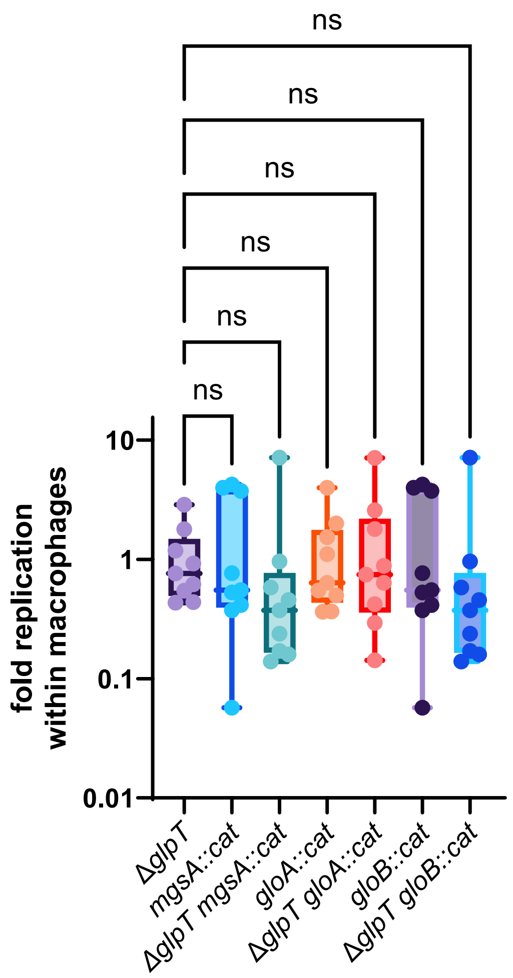

Figure S5 It remains unclear if GlpT contributes to replication in Raw264.7 cell in methylglyoxal-dependent and or -independent ways

The comparison of the replication rates for the *glpT* knock-out strain to *mgsA::cat*, *gloA::cat*, and *gloB::cat* deficient mutant strains does not have sufficient statistical power to discriminate whether *glpT*'s contributions to intramacrophage replication are methylglyoxal-dependent and -independent. The statistical significance was tested by an ordinary one-way ANOVA with Tukey correction. The lines indicate the median. n = 9 Raw264.7 macrophage infections, combined from three separate experiments.
