# Supplementary material for: Context-dependent change in the fitness effect of (in)organic phosphate antiporter glpT during Salmonella Typhimurium infection
Source: Nat Commun. 2025 Feb 24;16:1912. doi: 10.1038/s41467-025-56851-5 (PMC11850910; doi:10.1038/s41467-025-56851-5)
Supplement: Supplementary file 2 — Reporting Summary [file 41467_2025_56851_MOESM2_ESM.pdf]

nature portfolio

Corresponding author(s): Noemi Santamaria de Souza and Wolf-Dietrich Hardt

Last updated by author(s): Dec 3, 2024

Reporting Summary

Nature Portfolio wishes to improve the reproducibility of the work that we publish. This form provides structure for consistency and transparency in reporting. For further information on Nature Portfolio policies, see our Editorial Policies and the Editorial Policy Checklist.

Statistics

For all statistical analyses, confirm that the following items are present in the figure legend, table legend, main text, or Methods section.

n/a	Confirmed	
☐	☒	The exact sample size (*n*) for each experimental group/condition, given as a discrete number and unit of measurement
☐	☒	A statement on whether measurements were taken from distinct samples or whether the same sample was measured repeatedly
☐	☒	The statistical test(s) used AND whether they are one- or two-sided<br>*Only common tests should be described solely by name; describe more complex techniques in the Methods section.*
☒	☐	A description of all covariates tested
☐	☒	A description of any assumptions or corrections, such as tests of normality and adjustment for multiple comparisons
☒	☐	A full description of the statistical parameters including central tendency (e.g. means) or other basic estimates (e.g. regression coefficient) AND variation (e.g. standard deviation) or associated estimates of uncertainty (e.g. confidence intervals)
☐	☒	For null hypothesis testing, the test statistic (e.g. *F*, *t*, *r*) with confidence intervals, effect sizes, degrees of freedom and *P* value noted<br>*Give P values as exact values whenever suitable.*
☒	☐	For Bayesian analysis, information on the choice of priors and Markov chain Monte Carlo settings
☒	☐	For hierarchical and complex designs, identification of the appropriate level for tests and full reporting of outcomes
☒	☐	Estimates of effect sizes (e.g. Cohen's *d*, Pearson's *r*), indicating how they were calculated

Our web collection on statistics for biologists contains articles on many of the points above.

Software and code

Policy information about availability of computer code

Data collection	*Provide a description of all commercial, open source and custom code used to collect the data in this study, specifying the version used OR state that no software was used.*
Data analysis	*Provide a description of all commercial, open source and custom code used to analyse the data in this study, specifying the version used OR state that no software was used.*

For manuscripts utilizing custom algorithms or software that are central to the research but not yet described in published literature, software must be made available to editors and reviewers. We strongly encourage code deposition in a community repository (e.g. GitHub). See the Nature Portfolio guidelines for submitting code & software for further information.

Data

Policy information about availability of data

All manuscripts must include a data availability statement. This statement should provide the following information, where applicable:

- Accession codes, unique identifiers, or web links for publicly available datasets
- A description of any restrictions on data availability
- For clinical datasets or third party data, please ensure that the statement adheres to our policy

Relevant raw data will be made availabile via the ETH research collection.

Research involving human participants, their data, or biological material

Policy information about studies with human participants or human data. See also policy information about sex, gender (identity/presentation), and sexual orientation and race, ethnicity and racism.

Reporting on sex and gender	NA
Reporting on race, ethnicity, or other socially relevant groupings	NA
Population characteristics	NA
Recruitment	NA
Ethics oversight	NA

Note that full information on the approval of the study protocol must also be provided in the manuscript.

Field-specific reporting

Please select the one below that is the best fit for your research. If you are not sure, read the appropriate sections before making your selection.

☒ Life sciences ☐ Behavioural & social sciences ☐ Ecological, evolutionary & environmental sciences

For a reference copy of the document with all sections, see nature.com/documents/nr-reporting-summary-flat.pdf

Life sciences study design

All studies must disclose on these points even when the disclosure is negative.

Sample size	For in vitro experiments, three biological replica were used. For in vivo experiments at least five mice were included. The sample sizes were chosen based on prior data from our lab and in agreement with the local regulations and animal welfare offices.
Data exclusions	No data were excluded from the analysis.
Replication	All the experiments were performed at least twice, with all attempts at data replication being successful
Randomization	Mice were randomly allocated to different groups.
Blinding	Blinding was not performed as the investigators needed to identify the mouse cages oto determine the infections and or treatments.

Behavioural & social sciences study design

All studies must disclose on these points even when the disclosure is negative.

Study description	NA
Research sample	NA
Sampling strategy	NA
Data collection	NA
Timing	NA
Data exclusions	NA
Non-participation	NA
Randomization	NA

Ecological, evolutionary & environmental sciences study design

All studies must disclose on these points even when the disclosure is negative.

Study description	NA
Research sample	NA
Sampling strategy	NA
Data collection	NA
Timing and spatial scale	NA
Data exclusions	NA
Reproducibility	NA
Randomization	NA
Blinding	NA

Did the study involve field work? ☐ Yes ☒ No

Reporting for specific materials, systems and methods

We require information from authors about some types of materials, experimental systems and methods used in many studies. Here, indicate whether each material, system or method listed is relevant to your study. If you are not sure if a list item applies to your research, read the appropriate section before selecting a response.

Materials & experimental systems

n/a	Involved in the study	
☐	☒	Antibodies
☐	☒	Eukaryotic cell lines
☒	☐	Palaeontology and archaeology
☐	☒	Animals and other organisms
☒	☐	Clinical data
☒	☐	Dual use research of concern
☒	☐	Plants

Methods

n/a	Involved in the study	
☒	☐	ChIP-seq
☐	☒	Flow cytometry
☒	☐	MRI-based neuroimaging

Antibodies

Antibodies used	Anti-CSF1R (LuBio Science #BE0213),<br>Isogenic antibody control: IgG2a raised in rats (LuBio Science #BE0089),<br>Goat anti-human IgG Fcγ fragment specific AF647 Jackson ImmunoResearch Europe,<br>human anti-O12 antibody (hSTA5) was kindly gifted by the group of Prof. Antonio Lanzavecchia, Institute for Research in Biomedicine, Bellinzona, Switzerland
Validation	Commercial antibodies were validated by the manufacturer, whereas a fluorescence minus one control was utilised to establish the cutoff for distinguishing cells as either negative or positive for the antibody.

Eukaryotic cell lines

Policy information about cell lines and Sex and Gender in Research

Cell line source(s)	Raw264.7 macrophage cell line from a tumor in a male mouse induced with the Abelson murine leukemia virus, purchased from ATCC.
Authentication	*Describe the authentication procedures for each cell line used OR declare that none of the cell lines used were authenticated.*
Mycoplasma contamination	*Confirm that all cell lines tested negative for mycoplasma contamination OR describe the results of the testing for mycoplasma contamination OR declare that the cell lines were not tested for mycoplasma contamination.*

Commonly misidentified lines (See ICLAC register)	NA

Animals and other research organisms

Policy information about studies involving animals; ARRIVE guidelines recommended for reporting animal research, and Sex and Gender in Research

Laboratory animals	8- to 12-week old C57BL/6 mice (JAX:000664, The Jackson Laboratory) mice were held under specific pathogen-free (SPF) conditions at the ETH Phenomics Centre (EPIC) at ETH Zürich (light/dark cycle 12:12 h, room temperature 21±1 ̊C, humidity 50±10%). Germ-free were bred in flexible film isolators at the isolator facility at the EPIC facility.
Wild animals	NA
Reporting on sex	Mice were randomly assigned to experimental groups regardless of their sex.
Field-collected samples	NA
Ethics oversight	All animal experiments were reviewed and approved by the Kantonales Veterinäramt Zürich under the licenses ZH158/2019, ZH108/2022 and ZH109/2022, in compliance with the cantonal and Swiss legislation

Note that full information on the approval of the study protocol must also be provided in the manuscript.

Plants

Seed stocks	NA
Novel plant genotypes	NA
Authentication	NA

Flow Cytometry

Plots

Confirm that:

☒ The axis labels state the marker and fluorochrome used (e.g. CD4-FITC).

☒ The axis scales are clearly visible. Include numbers along axes only for bottom left plot of group (a 'group' is an analysis of identical markers).

☒ All plots are contour plots with outliers or pseudocolor plots.

☒ A numerical value for number of cells or percentage (with statistics) is provided.

Methodology

Sample preparation	GFP expression was analysed from murine feces using a plasmid-based PglpT-gfp reporter. In vivo samples were homogenized in PBS and 2 µg ml-1 of chloramphenicol (AppliChem) to inhibit additional protein synthesis and allow GFP proteins to fully mature. S. Tm was stained with a human anti-S. Tm O12 antibody (hSTA5; kindly gifted by Prof. Antonio Lanzavecchia, Institute for Research in Biomedicine, Bellinzona, Switzerland), and goat anti-human IgG AF647 antibody (Jackson ImmunoResearch Europe). Fluorescence was measured with a Cytoflex flow cytometer (Beckman Coulter) using the CytExpert software version 2.5.
Instrument	Beckman Coulter CytoflexS
Software	FlowJo V10, CytExpert software v.2.5
Cell population abundance	Approximatley 6% of acquired events stained positive for S. Tm O12.

Gating strategy

Gating strategy for S. Tm from feces using the primary antibody human anti-S. Tm O12 and the secondary antibody goat anti-human AF647. Including a fluorescence minus one control where the primary antibody was not added and a control S. Tm strain that does not express GFP.

☒ Tick this box to confirm that a figure exemplifying the gating strategy is provided in the Supplementary Information.
